# Focused ultrasound-mediated suppression of chemically-induced acute epileptic EEG activity

**DOI:** 10.1186/1471-2202-12-23

**Published:** 2011-03-06

**Authors:** Byoung-Kyong Min, Alexander Bystritsky, Kwang-Ik Jung, Krisztina Fischer, Yongzhi Zhang, Lee-So Maeng, Sang In Park, Yong-An Chung, Ferenc A Jolesz, Seung-Schik Yoo

**Affiliations:** 1 Department of Radiology, Brigham and Women's Hospital, Harvard Medical School, Boston, MA, USA; 2 The Semel Institute for Neuroscience and Human Behavior, David Geffen School of Medicine, University of California, Los Angeles, CA, USA; 3 Department of Physical Medicine & Rehabilitation, Hallym University Sacred Heart Hospital, Medical College of Hallym University, Anyang, Korea; 4 Institute of Catholic Integrative Medicine (ICIM), Incheon Saint Mary's Hospital, The Catholic University of Korea, Incheon, Korea

## Abstract

**Background:**

Epilepsy is a common neurological disorder, which is attributed to uncontrollable abnormal hyper-excitability of neurons. We investigated the feasibility of using low-intensity, pulsed radiation of focused ultrasound (FUS) to non-invasively suppress epileptic activity in an animal model (rat), which was induced by the intraperitonial injection of pentylenetetrazol (PTZ).

**Results:**

After the onset of induced seizures, FUS was transcranially administered to the brain twice for three minutes each while undergoing electroencephalographic (EEG) monitoring. An air-backed, spherical segment ultrasound transducer (diameter: 6 cm; radius-of-curvature: 7 cm) operating at a fundamental frequency of 690 KHz was used to deliver a train of 0.5 msec-long pulses of sonication at a repetitive rate of 100 Hz to the thalamic areas of the brain. The acoustic intensity (130 mW/cm^2^) used in the experiment was sufficiently within the range of safety guidelines for the clinical ultrasound imaging. The occurrence of epileptic EEG bursts from epilepsy-induced rats significantly decreased after sonication when it was compared to the pre-sonication epileptic state. The PTZ-induced control group that did not receive any sonication showed a sustained number of epileptic EEG signal bursts. The animals that underwent sonication also showed less severe epileptic behavior, as assessed by the Racine score. Histological analysis confirmed that the sonication did not cause any damage to the brain tissue.

**Conclusions:**

These results revealed that low-intensity, pulsed FUS sonication suppressed the number of epileptic signal bursts using acute epilepsy model in animal. Due to its non-invasiveness and spatial selectivity, FUS may offer new perspectives for a possible non-invasive treatment of epilepsy.

## Background

Epilepsy manifests developmental, cognitive, socioeconomic, and medical implications while the associated costs to society are staggering [1]. Anticonvulsant medications represent the first line of treatment for epilepsy. Although anti-epileptic/anti-ictal medications are readily available, approximately one third of patients are resistant to these pharmacological treatments [2]. To alleviate/treat intractable epilepsy with a localized origin, invasive neurosurgical approaches are adopted, such as surgical resection of the epileptogenic regions [3]. Subdural and epidural cortical stimulation, vagus nerve stimulation (VNS), and deep brain stimulation (DBS) have also been considered as viable treatment options [4]. However, these surgical approaches accompany inevitable risks associated with their invasiveness [5]. Therefore, a new and non-invasive treatment option is warranted to reduce or even extinguish epileptogenic activity.

Several non-invasive techniques are being tested for the suppression of epileptic activity [6]. For instance, transcranial magnetic stimulation (TMS) was suggested as a potential tool for the non-invasive treatment of epilepsy [7,8]. However, due to the inductive nature of magnetic stimulation, the area of modulation affected by the TMS is rather wide (on the order of several centimeters) and is limited to the cortical surface [9]. Transcranial direct current stimulation (tDCS) has also been introduced to suppress epileptic seizures through direct injection of electrical current into the brain [10]; nevertheless, it also lacks spatial specificity and suffers from a limited depth of penetration [6].

Recent advances in image-guided focused ultrasound (FUS) techniques allow for the non-invasive and spatially-accurate (on the order of millimeters) transcranial delivery of acoustic energy (in the form of mechanical and thermal energy) to a focused tissue region [11]. Several investigations of the effects of FUS on the *ex vivo *animal brain have revealed that ultrasound can temporarily modify the excitability of the neuronal tissue [12], which is possibly mediated by the regulation of ion channels without raising the local tissue temperature [13]. Ultrasound is also known to decrease cortical excitability, as it has been demonstrated by concurrent monitoring of visual evoked potentials in cats [14]. We recently demonstrated that the administration of low-intensity FUS (spatial-peak temporal average intensity; I_spta _< 165 mW/cm^2^) to a regional brain area, delivered in a train of pulses, modulated (*i.e*., excited or suppressed) neuronal excitability *in vivo *[15,16]. The modulation was achieved without altering the tissue temperature. By utilizing such a modulatory property of the pulsed sonication, especially to decrease the excitability, we were motivated to further examine if the FUS could reduce hyper-excitability of neural tissue based on a chemical kindling model of acute-stage epilepsy.

The goal of the study was to investigate the feasibility of using pulsed FUS to suppress epileptic neural activity, which was induced by the intraperitonial injection of pentylenetetrazol (PTZ) into rats. PTZ, a *gamma*-aminobutyric acid (GABA) receptor antagonist (particularly GABA_A_-receptor), has been extensively used in animal models to study acute-stage epilepsy [17]. An acute stage of epilepsy can be induced by a single dose of PTZ [18], which increases neuronal excitability across the entire brain volume with dominant hyper-excitability across the thalamus [19]. In this respect, we applied pulsed ultrasound to the brain to sonicate the entire thalamic area of rats with PTZ-induced acute epilepsy and measured subsequent electroencephalogram (EEG) activity to evaluate the degree of epileptic activity. Although the different thalamic subdivisions may differ in their roles in epilepsy [20], FUS in the present study was targeted to sonicate most of the thalamic area globally rather than its specific subdivisions. Therefore, sonication allows for the examination of the global effects of FUS on the thalamus and its potential role in the suppression of epileptic discharges. The results were compared to EEG activity observed in a group of PTZ-induced epileptic rats that did not receive sonication. Behavior of the animals after the treatment was also monitored, and the results were compared between the groups. In order to evaluate the safety of sonication itself, histological analysis was performed on the epilepsy-free animals to assess tissue or vascular damage at different time points after the sonication.

## Methods

### Study overview

All procedures were carried out in accordance with the ethical and safety rules set forth by the Institutional Animal Care and Use Committee of Harvard Medical School (protocol # 04608). Male Sprague-Dawley rats (280 ± 24 g prior to the epilepsy induction; *n *= 27) were randomly divided into three groups. Those in Group 1 (induction of acute epilepsy via PTZ injection) were treated with FUS sonication on the brain after PTZ-administration (*n *= 9: noted as 'PTZ(+)/FUS(+)'). This group allowed us to investigate the effects of sonication on an acute seizure model. Group 2 (PTZ injected rats without sonication; *n *= 9: noted as 'PTZ(+)/FUS(-)') provided a control condition to evaluate the EEG features of PTZ-induced epilepsy in the absence of sonication. Group 3 (*n *= 9) underwent sonication without the epilepsy induction (noted as 'PTZ(-)/FUS(+)') to provide another condition that was aimed to examine the presence of potential tissue damage imposed by the ultrasound. Group 3 allowed us to investigate the biological effects of FUS alone without the potential confounding effects associated with PTZ-induced neural damage. EEG measurements were not obtained from the non-epileptic animals in Group 3 since they were used solely to assess the extent of potential tissue damage imposed by sonication.

### Focused ultrasound sonication setup

An air-backed, spherical segment ultrasound transducer (diameter: 6 cm; radius-of-curvature: 7 cm) operating at a fundamental frequency of 690 KHz was used. This frequency is applicable for transcranial applications whereby the frequency range of 440 to 700 KHz has an optimal transmission gain through the *ex vivo *human skull [21,22]. The transducer was actuated by an electrical signal generated by a function generator (Agilent, Santa Clara, CA) which was concurrently amplified using a power amplifier (403LA, ENI Inc, Rochester, NY). The acoustic power generated for the given electrical signal as well as its spatial distribution were measured by a calibrated needle hydrophone (HNR500, ONDA, Sunnyvale, CA) mounted on a high-resolution 3-axis robotic stage (BiSlides, Velmex, Bloomfield, NY). The acoustic focus was roughly cigar-shaped and measured 3.5 mm in diameter and 6.2 mm in length at the full-width-at-half-maximum (FWHM) of the acoustic pressure field. We estimated the pressure amplitude after taking into account ultrasound attenuation through the rodent skull *in situ *(~ 87% of incoming sonication intensity; [23]).

The sonication parameters were controlled by software that changes the output pattern of the electrical signals from the function generator via a direct USB link (Sonomo, SensMed, Newton, MA). Based on our previous investigation into the suppressive effects of sonication on the rabbit brain [15,16], the following sonication parameters were used in the present study: 0.5 ms for the tone burst duration (TBD), 100 Hz for the pulse repetition frequency (PRF), and 130 mW/cm^2 ^(I_spta_) for acoustic intensity (expressed as power per unit area). The number of pulse trains determined the duration of sonication, and I_spta _was computed by a pulse intensity integral (*PII*) at the pulse repetition frequency. *PII *was estimated from the integral of the square of instantaneous pressure divided by the characteristic acoustic impedance [24]. This intensity value corresponded to 2.6 W/cm^2 ^in terms of spatial-peak pulse-average intensity (I_sppa_). The maximum peak negative pressure (^Max^P_n _in Pascal: Pa) at this parameter value was measured to be 0.27 MPa, which implies that damage to the tissue due to the pressure wave is highly unlikely [13]. Cavitation-related brain tissue damage, in the absence of air bubbles, is rare at pressures less than 40 MPa [25]. The mechanical index (MI), which is defined as the ^Max^P_n _of a longitudinal ultrasound wave propagating in a uniform medium divided by the square root of its center frequency, is used as the first-order limit to describe the safety of ultrasound devices. The MI of the present study (0.33) is also sufficiently low relative to the regulatory-limit for ultrasound procedures (*i.e*., 1.9 for all applications except ophthalmic (maximum 0.23); [26]). Additionally, our MI of 0.33 is less than the threshold for the blood-brain barrier (BBB) disruption in the presence of injected microbubbles (*e.g*., MI of 0.47; [27]). Without microbubbles to amplify the effects of acoustic pressure, the BBB is hardly disrupted, even at much higher acoustic intensities [27].

**Figure 1:**
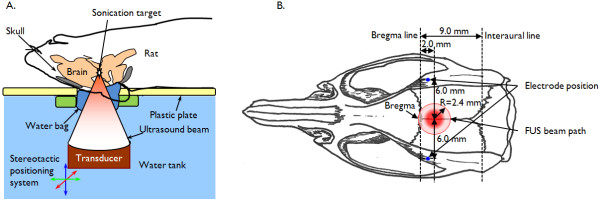
**A diagram of the experimental apparatus and the topographical arrangement of the sonication path and electrodes**. (A) The transducer was mounted on a 3-axis stereotactic positioning system and submerged in degassed water. The coordinates of the acoustic focus were controlled under the metric guidance of the stereotactic coordinates of the rat brain, which were marked on a plastic plate. After localization of the acoustic focus, the animal was laid supine on a plastic tray that was mounted above the system. The head was partially submerged in an oval hole that opened into a bag of degassed water in order to secure an uninterrupted beam profile from the transducer to the targeted tissue. (B) Schematic diagram of the positions of bilateral subdermal EEG electrodes (marked as small blue circles) relative to the area of the sonication beam path (marked as a red circular boundary) on the rat skull as well as to the sonication focus (marked as a solid red circle).

The FUS sonication apparatus is displayed in Figure 1A. The transducer was mounted on the 3-axis positioning system (UniSlides, Velmex, Bloomfield, NY) and was submerged in degassed water. The animal was laid supine on a plastic tray mounted above the system, and the head was partially submerged into an oval hole that opened into a bag of degassed water to secure an uninterrupted path from the transducer to the targeted tissue. To provide the same experimental stress conditions across all groups, the animals in Groups 2 and 3 underwent the same physical restraint method as did the epileptic animals in Group 1. The animal's head and body were gently restrained using tapes to prevent potential movement-related EEG artifacts. Prior to the animal experiment, the coordinates of the acoustic focus were positioned at the center of an oval hole under the metric guidance of the stereotactic coordinates of rats [28]. Since PTZ typically induces neuronal excitation across the whole brain volume with dominant hyper-excitability in the thalamic areas [19,29,30], the sonication focus was targeted to the thalamus (about 5 mm deep from the surface, along the midline, and 2 mm posterior to the bregma; cf. Figure 1B). Specifically, the sonication focus and the beam path were large enough to affect most of the thalamic areas.

### Animal preparation and pre-epilepsy EEG setup

**Figure 2:**
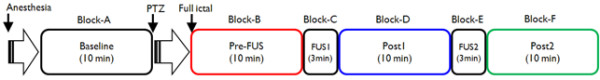
**Flowchart of the EEG acquisition and FUS sonication**. Block-A represents the baseline period. The baseline EEG was recorded for ten minutes after the EEG signals stabilized following the administration of anesthesia. Block-B (named as 'Pre-FUS') indicates the time-interval after observing significant evidence of ictal behavior (*e.g*., bilateral forepaw-twitches) and just before the first sonication. Block-C represents the three-minute period of the first sonication (named as 'FUS1'), and Block-E represents the second three-minute sonication interval (named as 'FUS2'). Block-D represents the time-interval after the first sonication (named as 'Post1'), and Block-F represents the time-interval after the second sonication (named as 'Post2').

Food and water were provided *ad libitum *to animals that were in a controlled day/night condition (12 hr/12 hr). All animals were anesthetized with an intraperitonial injection of xylazine (10 mg/kg) and ketamine (80 mg/kg). A pair of thin (~200 μm in diameter) Ag/AgCl electrodes (SWE Ives EEG Solution, Canada) was subdermally introduced into both left and right brain regions; the topographical position of these two electrodes was 6 mm lateral to the midline and 1 mm posterior to the bregma under the stereotactic guidance (small animal stereotaxic frame: SAS-4100, ASI instruments, Warren, MI; cf. Figure 1B; [28]). Since direct sonication of the electrode itself can potentially introduce significant confounding signals to the measured EEG and may also cause a rise in local tissue temperature due to the absorption of ultrasound energy (which is highly unlikely due to the use of a low acoustic energy of 130 mW/cm^2 ^I_spta_), the electrodes were positioned away from the incident sonication path as well as the sonication focus (cf. Figure 1B). Given the depth of the sonication focus (5 mm from the surface) and the relatively flat skull surface (perpendicular to the incident sonication beam), the radius of the sonication beam on the skull was calculated to be 2.4 mm by a trigonometric relationship of the transducer geometry (*i.e*., R = depth at the focus × tan(arcsin(half of outer diameter of the segmented transducer/radius-of-curvature)). The electrodes, placed subdermally, were positioned away from the sonication focus that was inside the brain. Another electrode was positioned at the tip of the ear as a ground electrode. The EEG signal was amplified (PowerLab 8/30, AD Instrument, CO) and recorded (LabChart 7, AD Instrument, CO) at a sampling rate of 1000 Hz. The EEG signal was recorded for ten minutes to establish the baseline condition (noted as 'Block-A' in Figure 2 and 'Baseline' in Figures 2 and 4).

### Induction of epilepsy and FUS sonication

45 mg/kg (based on animal weight) of PTZ prepared in 0.4 mL of normal saline was administered to the animals in Groups 1 and 2 via intraperitonial injection. EEG was subsequently acquired for ten minutes (noted as 'Block-B' or 'Pre-FUS' in Figure 2) after there was significant evidence of the epileptic behaviors (*e.g*., bilateral forepaw-twitches). The first dose of FUS sonication was then delivered to the animals for three minutes (noted as 'Block-C' or 'FUS1' in Figure 2). The EEG signals were measured after the first sonication for additional ten minutes (noted as 'Block-D' or 'Post1' in Figure 2). The second dose of FUS sonication was then administered to the animals (noted as 'Block-E' or 'FUS2' in Figure 2) for additional three minutes, followed by EEG-monitoring for another ten minutes (noted as 'Block-F' or 'Post2' in Figure 2). Upon the completion of the procedure, the animal was returned back to the cage for behavioral monitoring that was resumed on the following day.

### Behavioral monitoring

Behavioral monitoring was performed to evaluate the effects of sonication on epileptic behavior. The posture of the animal was evaluated for two weeks using the established Racine scoring system [31], and evaluation was performed at the same time on each day (*i.e*., between 10 am-11 am on Days 0-3, 5, 7, 10 and 14). During this period, the body weight was also monitored for excessive weight loss (*i.e*., more than 10% of original body weight).

### Histological analysis

The biological effect of sonication was examined from a separate group of rats (Group 3: *n *= 9) which did not undergo PTZ injection. The same experimental procedures were conducted to Group 3 as the other groups with the exception of PTZ injection. The acute effect of FUS sonication was examined from three animals that were sacrificed immediately after the procedure. The remaining six animals in Group 3 were allowed to survive after the FUS for either one week (*n *= 3) or three weeks (*n *= 3) to monitor any adverse behavioral changes associated with the procedure. The extracted brain was fixed with systemic circulation of formaldehyde (4% formaldehyde in phosphate buffered saline). For the preparation of the histological samples, the tissue was cut in the plane perpendicular to the sonication path. Haematoxylin and eosin (H&E) staining was used to assess potential hemorrhaging or microscopic tissue damages. The presence of DNA fragmental damage was probed by the terminal deoxynucleotidyl transferase-mediated dUTP-biotin nick end labeling (TUNEL) assay using the *in situ *cell death detection kit (Roche, Indianapolis, IN) developed using Cy2-conjugated streptavidin (1:500; Jackson Lavoratoried, West Grove, PA). The nucleus was counterstained with diamidino-2-phenylindole (DAPI). The slides were then examined under a light/fluorescence microscope (ELIPSE 80i, Nikon, Tokyo, Japan).

### EEG data analysis

We assessed both raw EEG and its theta-band activity. To analyze EEG data in the frequency domain, band-pass digital filtering was applied. The band-pass filter was implemented as a linear phase Finite Impulse Response (FIR) filter and was designed using the window method with a Kaiser window [32], giving pass and stop band ripples of less than 0.5%. The transition width of filtering was set to 20% of the cut-off frequency. The cut-off frequency was the frequency where the output amplitude falls to half the input amplitude (-6 dB). The cut-off frequency ranging from 4 to 8 Hz (*i.e*., theta-bands) was selected for spectral analysis since progressive increments of theta activity has been reported during PTZ-induced epilepsy [33,34]. It has also been suggested that theta-like slow waves are associated with epileptiform discharges [35]. Although the subdermal electrode was not generally susceptible to the facial or paw twitches associated with epileptic activity, the spurious signal fluctuations exceeding 200 μV were excluded from further analysis.

A qualitative assessment was confirmed by a detailed quantitative analysis of the observed epileptic spikes. An automated algorithm was used to detect and count the number of peaks in the raw EEG signal after thresholding the signal amplitude within each time block (A to F as described above and shown in Figure 2). The threshold was set to a value greater than 4.75 standard deviations from the individual baseline EEG activity (corresponds to the detection of deviant signal peaks with one-tailed probability < 10^-6^; assuming normal probability distribution in EEG noise pattern during the baseline state). This provided the ability to discriminate PTZ-induced epileptic bursts of EEG activity from standard baseline signal fluctuations. The number of detected peaks was counted in every one-minute segment and averaged across the monitoring period. Regarding the analysis of the theta-band EEG activity, the number of peaks exceeding the threshold was counted using the same algorithm as in the raw EEG analysis.

For statistical analyses, an independent two-sample *t*-test (one-tailed) was performed on the number of threshold-exceeding EEG peaks comparing the Group 1 and Group 2. The *t*-test was also employed for comparing Racine scores between the two groups. The number of threshold-exceeding EEG signal bursts was also compared within each animal. In this case, a paired *t*-test (one-tailed) was applied. In order to compare body weights of the animals between groups after the sonication periods, a repeated-measures ANOVA was performed while covarying for the individual body weight measured before the experiment.

## Results

### EEG data

**Figure 3:**
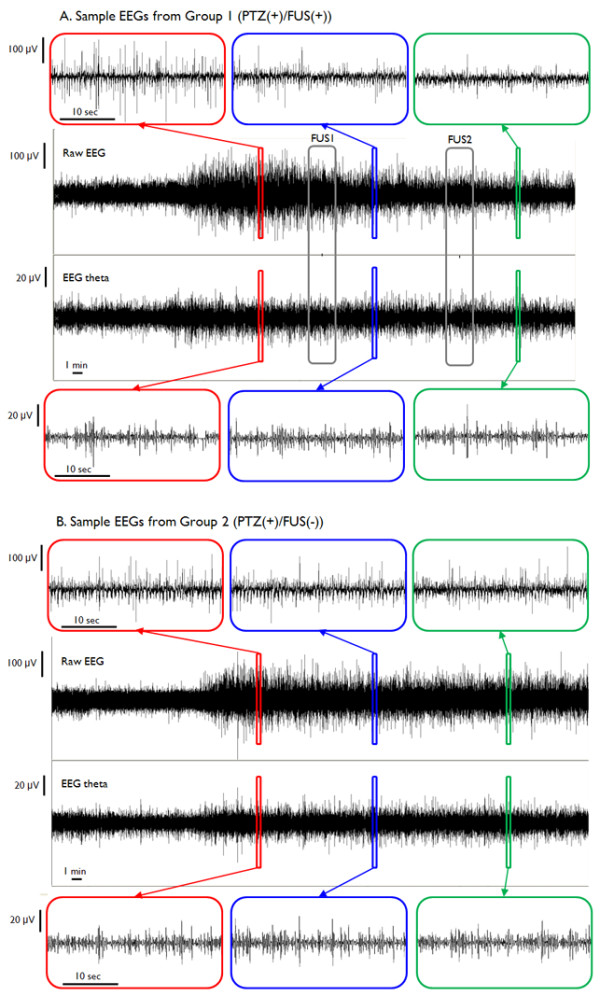
**The sample time-courses of EEG recordings from the test groups**. The representative example of EEG recordings from PTZ-induced epileptic rats (A) with sonication and (B) without sonication. In each EEG dataset, the upper signals represent unfiltered (raw) EEG data, and the lower signals show their corresponding theta-band activity. The insets of magnified windows represent EEG samples for 30 seconds in each highlighted time-window. Red boxes indicate an interval before the first sonication period, blue boxes indicate an interval between the first and the second sonication periods, and green boxes indicate an interval after the second sonication period. Note the changes in raw EEG spikes before, during, and after the sonication in the FUS-treated rat (see the upper red, blue and green boxes in (A)). The ictal activity during the pre-sonication period started to diminish along with each of the two sonication sessions (marked with gray boxes; FUS1-2) and was effectively suppressed after the second sonication (see the upper green box in (A)). Vertical scale bars indicate 100 μV in raw EEG signals and 20 μV in theta activity. Horizontal scale bars indicate a one-minute time scale (ten seconds for the insets).

**Figure 4:**
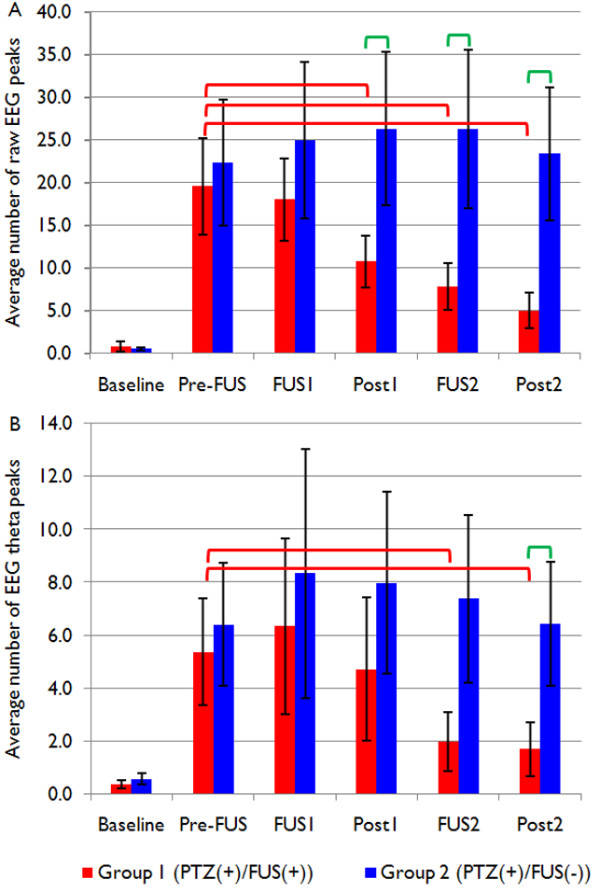
**Analysis of epileptic EEG signal bursts**. (A) Comparison of the average number of threshold-exceeding raw EEG peaks (greater than 4.75 standard deviations from the individual baseline activity of the raw EEG data) between the FUS-treated and untreated groups. (B) Comparison of the average number of EEG theta peaks exceeding the absolute magnitude above the threshold (4.75 standard deviations from the individual baseline theta activity) between the same two groups. Red bars indicate Group 1 (the FUS-treated PTZ group: PTZ(+)/FUS(+)), and blue bars indicate Group 2 (the untreated epileptic group: PTZ(+)/FUS(-)). As shown in both graphs, there were no significant differences between the two groups before the sonication. Red brackets indicate statistically significant differences (*p *< 0.05) within the FUS-treated group, and green brackets indicate statistically significant differences between the two groups. Error bars indicate standard errors of the mean.

Exemplary EEG data acquired from one animal within each group (Groups 1 and 2) are shown in Figure 3, whereby both unfiltered EEG recordings and theta-band activity are displayed. Prior to the PTZ injection, there was no apparent detection of epileptic EEG signal bursts (see 'Baseline' in Figure 4). Within approximately ten minutes after the administration of PTZ, epileptic signal bursts were observed in their EEG recordings (see red boxes in Figure 3 and 'Pre-FUS' in Figure 4). As demonstrated in Figure 3A (see the upper blue box compared to the upper red box), the number of EEG bursts in the FUS-treated rat appeared to be reduced after the first sonication (noted as 'FUS1'), but remained steady in the epileptic rat that did not receive the sonication (cf. Figure 3B and Figure 4A). After the second sonication (noted as 'FUS2'), the number of bursts was further reduced compared to the number obtained during and after the first sonication (see the upper green box compared to the previous red and blue ones in Figure 3A), whereas the control epileptic rat still showed a greater number of bursts throughout the entire monitoring period (cf. Figure 3B and Figure 4A). The EEG signals in the theta-bands showed similar trends as the unfiltered signals (cf. Figure 4B).

### Between-group effect

Prior to the injection of PTZ, the EEG recordings did not show any significant differences between the two PTZ groups (t(16) = 0.430, *n.s*.). After the PTZ-injection, both groups manifested a dramatic increase in the number of epileptic signal bursts, without any significant group differences (t(16) = -0.321, *n.s*.). Immediately after the first sonication (*i.e*., 'Post 1'), however, the number of epileptic EEG bursts decreased in the sonicated group (t(16) = -1.74, *p *< 0.05). As shown in Figure 4A, this suppressive effect (note the significant differences indicated by green brackets in Figure 4A) was maintained during and after the second sonication ('FUS2': t(16) = -2.03, *p *< 0.05; 'Post2': t(16) = -1.72, *p *< 0.05).

As for the analysis of EEG theta activity, there were no significant differences between the two groups prior to the first sonication ('Baseline': t(16) = -0.754, *n.s*.; 'Pre-FUS': t(16) = -0.355, *n.s*.). However, there were significant reductions in the number of EEG theta bursts in the sonicated group after the second sonication ('Post2': t(16) = -1.98, *p *< 0.05; see the green bracket in Figure 4B). Compared to the second sonication, the first sonication did not have any significant impact on reducing the number of EEG bursts in the theta-bands ('Post1': t(16) = -0.790, *n.s*.).

### Within-group effect

As shown in Figure 4A (see red brackets), the number of epileptic EEG bursts within the FUS-treated group was significantly reduced after the first sonication period ('Post1': t(8) = 2.26, *p *< 0.05) compared to the pre-sonication period ('Pre-FUS'). The degree of reduction was even greater during and after the second sonication period (up to 74.5%; 'FUS2': t(8) = 1.91, *p *< 0.05; 'Post2': t(8) = 2.58, *p *< 0.05). In the FUS-treated group, the number of epileptic signal bursts in the raw EEG data was further reduced after the second sonication compared to the number observed during and after the first sonication ('FUS1' vs. 'Post2': t(8) = 2.73, *p *< 0.05; 'Post1' vs. 'Post2': t(8) = 2.55, *p *< 0.05). Compared to the pre-sonication period, the number of threshold-exceeding EEG theta peaks was significantly reduced during (63.0% reduction) and after (up to 68.5% reduction) the second sonication ('FUS2': t(8) = 2.81, *p *< 0.05; 'Post2': t(8) = 3.14, *p *< 0.01). In contrast, the number of detected signal bursts within the unsonicated epileptic group for both raw and theta EEG activity remained constant during the entire monitoring period (compared to the period of full-fledged epileptic EEG bursts, which corresponds to 'Pre-FUS'; all *n.s*.). We observed that the occurrence of threshold-exceeding raw EEG bursts after the two sonication periods did not completely recover to the pre-PTZ injection state (*i.e*., the baseline period; t(8) = -2.83, *p *< 0.05), whereas the number of threshold-exceeding EEG theta bursts returned back to the state prior to epilepsy induction (t(8) = -1.55, *n.s*.).

### Behavioral and histological data

**Figure 5:**
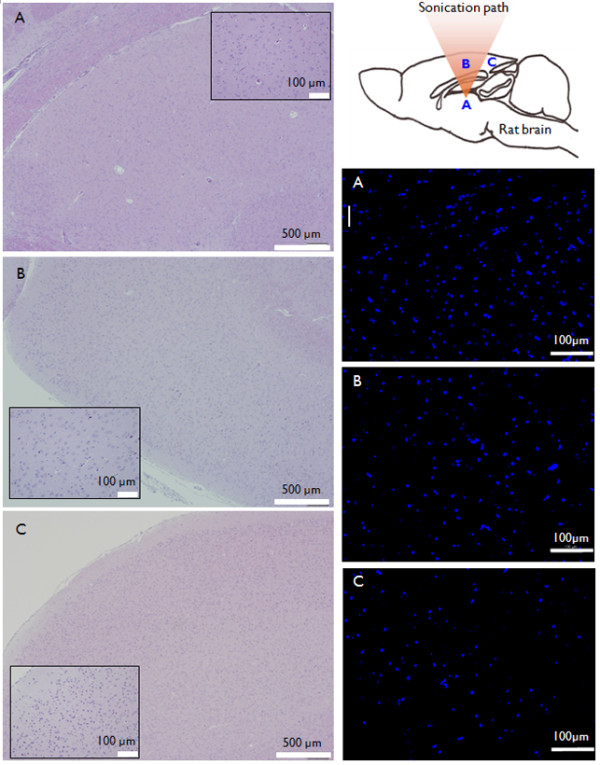
**Examples of histology from the sonicated brain area**. Exemplary histological data obtained from Group 3 (PTZ(-)/FUS(+)). (Left column) H&E staining results and (Right column) TUNEL staining results (DAPI in blue, apoptotic cell in green) from (A) a sonicated thalamic site, (B) the cortex above the sonicated thalamus in the FUS beam path, and (C) an unsonicated posterior cortex. Note the absence of apoptotic DNA-damaged cells in all of the examined locations.

The Racine scores that were measured on the day after the experiment were significantly lower in the FUS-treated group than those of the unsonicated epileptic group (t(15) = -2.41, *p *< 0.05; Group 1: 0.33 ± 0.18, Group 2: 1.13 ± 0.30). This distinction disappeared while the Racine scores indicated the non-epileptic state after Day 2 (t(15) = -1.75, *n.s*.; Group 1: 0 ± 0, Group 2: 0.13 ± 0.13). It suggests that both groups recovered from the acute epilepsy induction by PTZ. The body weight of the animal, which is an indicator of the long-term severity of epilepsy, was shown to be indifferent between the sonicated and unsonicated group (F(1,14) = 0.617, *n.s*.). Histological analysis performed on Group 3, indicated that no detectable tissue damage was observed after the FUS sonication. Based on TUNEL staining, there was no apparent indication of DNA fragmentation in the brain tissue located at or near the sonicated sites (cf. Figure 5) throughout the monitoring period (up to three weeks). To further examine the presence of apoptosis, the direct assessment of caspases (or cysteine-aspartic proteases) activity is needed [36]. Visual and histological (H&E) inspection did not show any observable brain tissue damage in close proximity to the electrodes, which were located subdermally above the skull.

## Discussion

Our results reveal that low-intensity, pulsed FUS sonication suppressed the number of epileptic signal bursts observed in EEG recordings after the induction of acute epilepsy via intraperitoneal injection of PTZ. The presence of the suppressive effect was found in terms of the number of epileptic EEG spikes from the analysis of the unfiltered (Figure 4A) and theta-band (Figure 4B) EEG activity. FUS-mediated reduction of epileptic EEG activity was most notably observed in the theta band. EEG theta activity has also been consistently reported to have a positive correlation with the level of epilepsy [33-35]. Thus, our findings may offer more information with regard to the possible mechanisms involved in the reduction of epileptic activity (for example, the region-specific efficacy of FUS and its manifestation in theta band activity). The assessment of EEG patterns associated with sonication, in the absence of induced epilepsy, will offer rich information on the excitatory or inhibitory influence of FUS on neural circuitry, and it will provide more information on the applicability of FUS to non-pathological conditions. We also found that the second sonication session further enhanced the suppressive effect beyond that of the first sonication session. Based on the analysis of the Racine scores, it consistently appears that the FUS-treated group recovered from an epileptic state more quickly than the unsonicated group.

Taken together, these findings suggest that transcranial FUS sonication provided a significant suppressive effect on PTZ-induced epileptic activity in rats. These observations are in good agreement with previous studies on the temporary suppression of spontaneous activity in the excised crayfish ventral nerve cord [37] and on the suppression of visual activity in cats mediated through insonication of non-focused ultrasound [14]. Although the inferior colliculus of rats is responsive to ultrasound [38] and can even induce audiogenic seizures [39,40], our observations are unlikely to be associated with the auditory responsiveness of the inferior colliculus of rats to ultrasound frequencies. This is because the ultrasound frequency of the present study (*e.g*., 690 KHz) was far greater than the audible range (applicable to rodents, approximately 30 to 70 KHz) of ultrasound frequencies in which the maximal responsiveness of the inferior colliculus of rats was observed [38]. Generally, the rodent species used in this study can process ultrasound up to approximately 80 KHz [41-43].

Since epileptic activity is caused by abnormally excessive or synchronous neural activity in the brain [44], and synaptic contacts could potentially be disrupted by ultrasound waves [45], FUS sonication might reduce the propagation of epileptic discharges across the brain. Alternatively, a different hypothesis can be put forth to explain our findings: FUS sonication may have caused a reduction in epileptic EEG activity by regulating thalamic GABAergic inhibitory neurons implicated in epilepsy [46]. Evaluation of the extracellular neurotransmitter levels (such as GABA) may offer useful information to clarify some of these hypotheses through the use of microdialysis techniques that assay various types of neurotransmitters directly from the brain [47].

Although little is known about the detailed mechanism underlying FUS-mediated neuro-modulation, it has long been reported that ultrasound can significantly affect the neurophysiology of *in vitro *local neural circuitry [48,49]. Gavrilov et al. [12] reported that the main effect of FUS in stimulating neural structures is due to mechanical force that could produce alterations in membrane potential, thus resulting in the stimulation of neural structures. It has also been proposed that ultrasound sonication may influence membrane fluidity, turbidity and permeability [50,51]. Accordingly, the activity of ion-channels or receptors on the membrane can be influenced by ultrasound sonication [49], and the trans-membrane concentrations or passage of ions or neurotransmitters can be subsequently altered. FUS-mediated structural alterations in soma/axonal/dendritic connections may also have attributed to our findings and thus requires further investigation.

It has been consistently reported that ultrasound sonication activates voltage-gated Na^+ ^and Ca^2+ ^channels [13] and that a FUS-mediated mechanical force can activate several mechano-sensitive ion channels, allowing cation entry [52-55] and resulting in alterations in membrane potential [12]. Therefore, FUS-mediated dysfunction of functional molecules, such as cell membrane transporters that are sensitive to trans-membrane ion-concentrations, may lead to biochemically altered states in the sonicated area. For example, activation of the serotonin transporter (SERT) is modulated by the trans-membrane gradient of Na^+ ^and K^+ ^[56], and trans-membrane ion concentrations are potentially altered by FUS sonication. Consequently, abnormal SERT activity, possibly by FUS sonication, may actuate a change in the extracellular level of serotonin (5-HT).

In terms of biological safety, it is noteworthy that ultrasound sonication can potentially generate free radicals [57,58]. For example, ultrasound sonication can decompose water into hydrogen and hydroxyl radicals [59]. These free radicals, although short-lived, are extremely unstable and can react easily with other surrounding biological molecules, possibly resulting in tissue damage and inflammatory response [60]. However, these free radicals are typically produced at high acoustic intensities that are associated with cavitation [61]. Since the current study uses an acoustic intensity much lower than those that produce cavitation and free radicals, sonication in the present study is unlikely to adversely affect the brain tissue.

As shown in the histological results (cf. Figure 5), the sonication employed in the present study did not cause any inadvertent biological damage to the target region. The intensity of sonication used in the present study was 130 mW/cm^2 ^(I_spta_), which is far less than the upper regulatory limit for non-obstetric ultrasound imaging (720 mW/cm^2^; [62]). It has been reported that when a short duration of sonication (5 sec) is used, ultrasound intensity up to 430 W/cm^2 ^(at 936 KHz) can be applied without inducing mechanical damages to the brain tissue [63]. The MI of the present study was 0.33, which is sufficiently within the range of safety guidelines (*i.e*., 1.9; [26]). Collectively, our sonication parameters are all within the range of safety guidelines for clinical ultrasound imaging and demonstrate a significant reduction of epileptic activity characterized in EEG and behavioral monitoring.

The present study has several technical limitations to overcome. First of all, since the intraperitoneal injection of PTZ elicits hyper-excitability over the distributed regions of the brain, region-specific anti-epileptogenic effects of FUS were not demonstrated. In order to examine the utility of FUS in suppressing region-specific epileptogenic activity, a regional chemical kindling model such as an intracortical injection of kainic acid (KA) can be adopted to induce focal epileptic lesions in an animal model. KA induces nonconvulsive status epilepticus, followed by the chronic occurrence of spontaneous recurrent seizures and massive hippocampal damage [64-66]. The regional application of FUS to a KA-kindled epileptogenic focus for probing its potential utility in the treatment of chronic focal epilepsy constitutes one of our future subjects of investigation.

Another technical limitation of the study is the spatial error introduced while positioning the sonication focus. There are several sources that can contribute to potential spatial error while targeting the sonication focus to the thalamic area. These sources include the inherent mechanical repositioning error of the mechanical 3-axis stage that mounted the transducer as well as spatial error associated with acoustic field distortion during transcranial FUS application. Since these sources typically introduce errors that are significantly smaller than the acoustic focus, the major source of spatial error during positioning of the focus can be traced to the use of an external anatomical landmark, *i.e*., the ear canal and associated inter-aural lines, during stereotactic positioning of the animal with respect to the sonication apparatus. Based on the work by Rubins et al. [67], the potential spatial error associated with the procedure can be estimated to be on the order of 0.5 mm, which is approximately 15% of the short-axis diameter of the FUS focus (3.5 mm in diameter). The characterization of the exact location and size of the sonication focus in the brain would clearly improve the spatial accuracy of sonication delivery. The use of magnetic resonance imaging (MRI) enables an elaborate spatial guidance system for the application of focused acoustic energy to a defined anatomical location [11,68,69]. For example, an MRI-compatible stereotactic positioning system [70,71] would allow users to track the coordinates of the sonication focus. Localization of the sonication focus can also be accomplished by the guidance of acoustic radiation force impulse (ARFI) imaging which can visualize the degree of acoustic force imposed on tissues without the generation of heat [72-74].

It is also noteworthy that FUS can elicit neuronal stimulation with different sets of FUS-parameters (*i.e*., TBD = 50 msec, PRF = 10 Hz in rabbits or TBD = 0.4 msec, PRF = 1500 Hz in rats, and both achieved at a higher acoustic intensity of I_spta _4~6 W/cm^2^; unpublished data). Therefore, it is reasonable to predict that FUS could further exacerbate neuronal hyperactivity in epilepsy. Accordingly, further studies on parameter-dependent efficacy of the method and careful selection of the sonication parameters are needed to develop appropriate treatment guidelines.

FUS-mediated region-specific functional neuro-modulation promises new, powerful ways to study brain function and brain-behavior relations. As a result, we anticipate that this technique may influence the development of new modalities for neurotherapeutic treatments across a wide clinical range. For instance, neurological conditions that are associated with subcortical structures (*i.e*., pain and movement disorders related to abnormalities in the thalamus and elements of the limbic system) may potentially benefit from FUS due to its ability to reach deep brain regions in a non-invasive way. Similarly, the modulatory effects of FUS can be utilized to modify aberrant brain activity and neurotransmission associated with various psychiatric conditions, such as depression or post-traumatic stress disorder.

## Conclusions

In summary, our findings provide compelling evidence that FUS sonication holds promise as an elegant non-invasive therapeutic tool to suppress epileptic activity. Since FUS-mediated transcranial thermal ablation of human brain tumors [75] and functional neurosurgical applications using targeted lesions in the thalamus [76] have already been accomplished using a commercially-available transcranial FUS prototype, the translation of the technique to human application would not encounter significant technical barriers. Further refinement of the sonication parameters and subsequent exploration of selective functional neuro-modulation will be needed to disseminate the technique across a wide range of research and clinical fields.

## Authors' contributions

BM carried out sonication experiment using animal model of acute epilepsy, conducted the data analysis and prepared the manuscript. KJ and YZ participated in the development of protocols for animal acute epilepsy model, and drafted the manuscript. KF prepared the histological sections while IM, SP, and YC performed the immunohistological analysis. AB participated in the design of the study and helped to draft the manuscript. SY conceived of the study, and participated in its design and coordination as well as in the preparation of the manuscript. All authors read and approved the final manuscript.

## Authors' information

Author AB is a founder and stockholder of Brainsonix, a California Corporation. The remaining authors have no conflicts of interest.
